# The analgesic efficacy of paravertebral block for percutaneous nephrolithotomy: A meta-analysis of randomized controlled studies

**DOI:** 10.1097/MD.0000000000017967

**Published:** 2019-11-27

**Authors:** Xiaoyu Tan, Donglin Fu, Wubing Feng, Xiangqi Zheng

**Affiliations:** a Department of Urology, The Fourth People's Hospital of Chongqing; b Department of Critical Care Medicine, Chongqing General Hospital, China.

**Keywords:** paravertebral block, analgesic efficacy, percutaneous nephrolithotomy, randomized controlled trials

## Abstract

**Introduction:**

The analgesic efficacy of paravertebral block for percutaneous nephrolithotomy remains controversial. We conduct a systematic review and meta-analysis to explore the analgesic efficacy of paravertebral block for patients with percutaneous nephrolithotomy.

**Methods:**

We have searched PubMed, EMbase, Web of science, EBSCO, and Cochrane library databases, and randomized controlled trials (RCTs) assessing analgesic efficacy of paravertebral block for percutaneous nephrolithotomy are included in this meta-analysis.

**Results:**

Five RCTs are included in the meta-analysis. Overall, compared with control group after percutaneous nephrolithotomy, paravertebral block is associated with the decrease in analgesic consumption (standard mean difference (Std. MD) = −1.55; 95% confidence interval (CI) = −2.18 to −0.92; *P* < .00001) and additional analgesics (risk ratio (RR) = 0.17; 95% CI = 0.07 to 0.44; *P* = .0003), prolonged time to first analgesic requirement (Std. MD = 1.51; 95% CI = 0.26 to 2.76; *P* = .02). There is no statistical difference of adverse events including nausea or vomiting (RR = 0.51; 95% CI = 0.11 to 2.35; *P* = .38), or itching (RR = 0.69; 95% CI = 0.26 to 1.81; *P* = .45) between 2 groups.

**Conclusions:**

Paravertebral block is effective for pain control after percutaneous nephrolithotomy.

## 1 Introduction

Percutaneous nephrolithotomy is widely used to treat staghorn stones and kidney stones. It remains the gold standard for the treatment of renal stone because of the less invasion and morbidity compared to open surgery.^[1–3]^ However, percutaneous nephrolithotomy can result in the distension of pelvicalyceal system and postoperative pain due to the inserted nephrostomy.^[4–6]^ Many methods such as systemic opioids, non-steroidal anti-inflammatory drugs, and epidural analgesic are developed to alleviate this pain and reduce the complications, hospitalization, and costs.^[7–9]^ However, these methods may cause serious adverse effects such as respiratory depression, sedation, nausea, vomiting, constipation, and renal problems.^[10,11]^

Paravertebral block is reported to be a successful regional method for pain relief in various surgeries.^[12,13]^ It can produce a unilateral, somatosensory and sympathetic block by the injection of local anesthetics into the paravertebral space containing thoracic spinal nerves and branches.^[14,15]^ Previous studies confirmed its analgesic efficacy in thoracotomy, breast surgery, and abdominal surgery.^[16,17]^ One RCT reports that ultrasound-guided thoracic paravertebral block is revealed to reduce postoperative opioid consumption and pain scores after percutaneous nephrolithotomy.^[18]^

Current evidence is insufficient for routine clinical use of paravertebral block for percutaneous nephrolithotomy, and several studies have investigated the efficacy and safety of paravertebral block for these patients.^[18–20]^ This systematic review and meta-analysis of RCTs aims to assess the analgesic efficacy of paravertebral block for percutaneous nephrolithotomy.

## 2 Materials and methods

This meta-analysis is conducted based on the Preferred Reporting Items for Systematic Reviews and Meta-analysis statement and Cochrane Handbook for Systematic Reviews of Interventions.^[21,22]^ We do not need ethical approval or patient consent, because all analyses are based on previous published studies.

### 2.1 Literature search and selection criteria

We have systematically searched several databases including PubMed, EMbase, Web of science, EBSCO and the Cochrane library from inception to May 2019, and use the following keywordsparavertebral block, and percutaneous nephrolithotomy. The inclusion criteria are as follows:

1. study design is RCT,
2. patients undergo percutaneous nephrolithotomy, and
3. intervention treatments are paravertebral block vs no intervention (or intravenous analgesic).

### 2.2 Data extraction and outcome measures

Some baseline information is extracted from the original studies, and they include first author, number of patients, age, weight, duration of anesthesia, and detail methods in 2 groups. Data are extracted independently by 2 investigators, and discrepancies are resolved by consensus. We have contacted the corresponding author to obtain the data when necessary.

The primary outcome is analgesic consumption. Secondary outcomes include additional analgesics, time to first analgesic requirement, nausea and vomiting, itching.

### 2.3 Quality assessment in individual studies

We evaluate the methodological quality of each RCT using the Jadad Scale which has 3 evaluation elements: randomization (0-2 points), blinding (0-2 points), dropouts, and withdrawals (0-1 points).^[23]^ The score of Jadad Scale varies from 0 to 5 points. Jadad score≤2 indicates low quality, while Jadad score≥3 suggests high quality.^[24]^

### 2.4 Statistical analysis

We assess standard mean difference (Std. MD) with 95% confidence interval (CI) for continuous outcomes, and risk ratio (RR) with 95% CIs for dichotomous outcomes in this meta-analysis. Heterogeneity is evaluated using the *I*^2^ statistic, and *I*^2^ > 50% indicates significant heterogeneity.^[25,26]^ The random-effects model is used for all meta-analysis. Sensitivity analysis is performed to detect the influence of a single study on the overall estimate via omitting 1 study in turn or performing the subgroup analysis. Publication bias is not assessed if there are the limited number (<10) of included studies. Results are considered as statistically significant for *P* < .05. All statistical analyses are performed using Review Manager Version 5.3 (The Cochrane Collaboration, Software Update, Oxford, UK).

## 3 Results

### 3.1 Literature search, study characteristics and quality assessment

**Figure 1:**
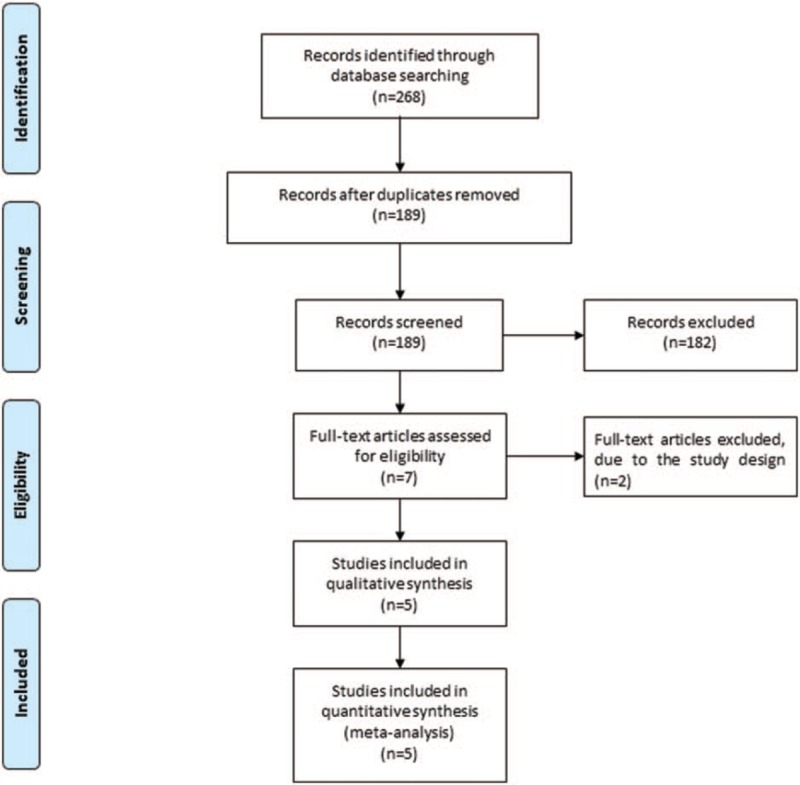
Flow diagram of study searching and selection process.

Figure 1 shows the detail flowchart of the search and selection results. 268 potentially relevant articles are identified initially, and 5 RCTs are finally included in the meta-analysis.^[7,10,18–20]^

The baseline characteristics of 5 included RCTs are shown in Table 1. These studies are published between 2013 and 2018, and the total sample size is 256. Four studies report 0.25% or 0.5% bupivacaine for paravertebral block ^[7,10,18,19]^ and the remaining RCT reports 0.5% bupivacaine plus 1 μg/kg of clonidine for paravertebral block.^[20]^ One RCT involves intravenous tramadol at the dose of 1 mg/kg in the control group.^[19]^

**Table 1:**
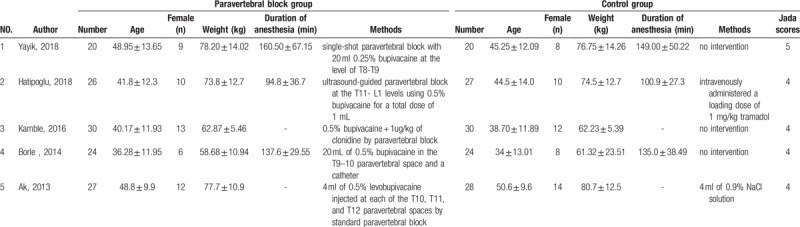
Characteristics of included studies.

Four RCTs report analgesic consumption and additional analgesics,^[7,10,18,19]^ 2 RCTs report the time to first analgesic requirement,^[7,10]^ 3 RCTs report nausea and vomiting,^[7,10,19]^ and 2 RCTs report itching.^[7,18]^ Jadad scores of the 5 included studies vary from 3 to 5, and they all have high-quality based on the quality assessment.

### 3.2 Primary outcome: analgesic consumption

**Figure 2:**
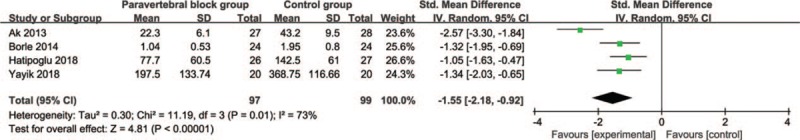
Forest plot for the meta-analysis of analgesic consumption.

The random-effect model is used for the analysis of analgesic consumption. The results find that compared to control group, paravertebral block results in significantly decreased analgesic consumption after percutaneous nephrolithotomy (Std. MD = −1.55; 95% CI = −2.18 to −0.92; *P* < .00001), with significant heterogeneity among the studies (*I*^2^ = 73%, heterogeneity *P* = .01, Fig. 2).

### 3.3 Sensitivity analysis

There is significant heterogeneity for the analgesic consumption. As shown in Figure 2, the study ^[7]^ shows the result that is completely out of range of the others and probably contributes to the heterogeneity. After excluding this study, the results suggest that paravertebral block can also reduce the analgesic consumption after percutaneous nephrolithotomy (Std. MD = −1.22; 95% CI = −1.58 to −0.86; *P* < .00001). No evidence of heterogeneity is observed among the remaining studies (*I*^2^ = 0%).

### 3.4 Secondary outcomes

**Figure 3:**
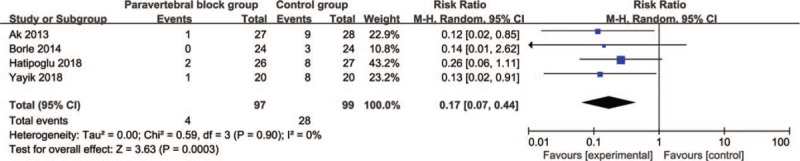
Forest plot for the meta-analysis of additional analgesics.

**Figure 4:**
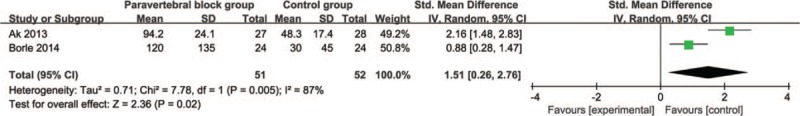
Forest plot for the meta-analysis of time to first analgesic requirement.

**Figure 5:**
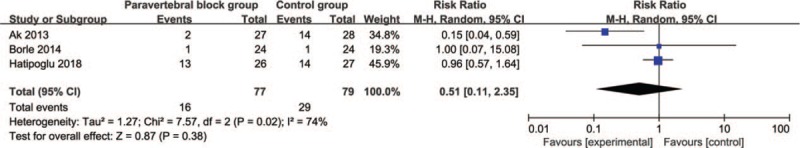
Forest plot for the meta-analysis of nausea and vomiting.

**Figure 6:**
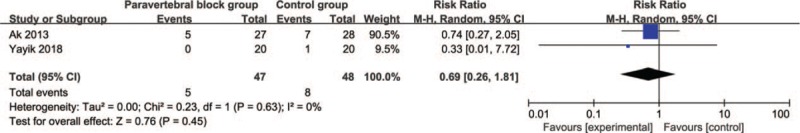
Forest plot for the meta-analysis of itching.

In comparison with control intervention following percutaneous nephrolithotomy, paravertebral block is associated with decreased additional analgesics (RR = 0.17; 95% CI = 0.07 to 0.44; *P* = .0003; Fig. 3), and prolonged time to first analgesic requirement (Std. MD = 1.51; 95% CI = 0.26 to 2.76; *P* = .02; Fig. 4), but has no substantial impact on nausea or vomiting (RR = 0.51; 95% CI = 0.11 to 2.35; *P* = .38; Fig. 5), or itching (RR = 0.69; 95% CI = 0.26 to 1.81; *P* = .45; Fig. 6).

## 4 Discussion

Percutaneous nephrolithotomy is prominent in the treatment of urinary system stone diseases,^[27–29]^ but the postoperative pain commonly occurs after percutaneous nephrolithotomy, and leads to increased hospitalization times, reduced quality of life, development of chronic pain, and some complications such as thromboembolic and pulmonary complications, respiratory depression, circulatory failure, disruption of bowel functions, urine retention, nausea, vomiting, and sleep disorders.^[30,31]^

Paravertebral block has proved the capability of postoperative pain control in various surgical patients, and shows no obvious impact on motor blockade, bowel movements, or hemodynamic balance.^[7,32,33]^ The analgesic fluid injected into thoracic paravertebral space may stay in the place of injection, spread towards the neighboring epidural space, and paravertebral space.^[34]^ Our meta-analysis suggests that paravertebral block can substantially reduce analgesic consumption, additional analgesics, and prolong the time to first analgesic requirement compared to control intervention after percutaneous nephrolithotomy, and there is similar incidence of nausea, vomiting, and itching between 2 groups.

In addition, 4 included RCTs report that pain scores in paravertebral block group after the surgery are found to be significantly lower than those in control group.^[7,10,18,19]^ Paravertebral block can provide the unilateral blockade of 4 or 5 thoracic dermatomes.^[35]^ Ultrasound is found to reduce the risk of complications such as nerve damage.^[36,37]^ Previous study revealed that a single level or 2 different levels of ultrasound-guided paravertebral block may provide no significant difference of spread in the paravertebral region.^[37]^ Regarding the sensitivity analysis, there is significant heterogeneity for the primary outcome, and no heterogeneity is observed after excluding the study conducted by Ak et al.^[7]^ This heterogeneity may be caused by different concentration of bupivacaine and injection levels of paravertebral block.

Several limitations exist in this meta-analysis. Firstly, our analysis is based on only 5 RCTs, and more RCTs with large sample size should be conducted to explore this issue. Next, there is significant heterogeneity, different concentration, and combination of bupivacaine, injection levels of paravertebral block may lead to this heterogeneity. Finally, some unpublished and missing data may result in some bias to the pooled effect.

## 5 Conclusion

Paravertebral block is effective to improve pain control after percutaneous nephrolithotomy.

## Author contributions

**Methodology:** Xiangqi Zheng.

**Writing – original draft:** Xiangqi Zheng.

**Writing – review & editing:** Xiangqi Zheng.
